# Identifying risk factors for clinical Lassa fever in Sierra Leone, 2019–2021

**DOI:** 10.1017/S095026882400164X

**Published:** 2024-11-20

**Authors:** Daniel Juma Sama, Najmul Haider, Javier Guitian, Abdinasir Yusuf Osman, Francine Ntoumi, Alimuddin Zumla, Richard Kock, Rashid Ansumana

**Affiliations:** 1 School of Public Health, Njala University, Bo City, Sierra Leone; 2 Department of Pathobiology and Population Sciences, The Royal Veterinary College, Hatfield, Hertfordshire, AL9 7TA, UK; 3 School of Life Sciences, Faculty of Natural Sciences, Keele University, Staffordshire ST5 5BG, UK; 4 Ministry of Health, Mogadishu, Somalia; 5 Congolese Foundation for Medical Research, Brazzaville, Republic of Congo; 6 Institute for Tropical Medicine, University of Tübingen, Tübingen, Germany; 7 Division of Infection and Immunity, Centre for Clinical Microbiology, University College London, London, UK; 8 National Institute of Health Biomedical Research Centre, University College London Hospitals NHS Foundation Trust, London, UK

**Keywords:** Lassa fever, Lassa fever virus, *Mastomys natalensis*, risk factors, rodents, Sierra Leone

## Abstract

Lassa fever (LF) virus (LASV) is endemic in Sierra Leone (SL) and poses a significant public health threat to the region; however, no risk factors for clinical LF have been reported in SL. The objective of this study was to identify the risk factors for clinical LF in an endemic community in SL. We conducted a case–control study by enrolling 37 laboratory-confirmed LF cases identified through the national LF surveillance system in SL and 140 controls resided within a one-kilometre radius of the case household. We performed a conditional multiple logistic regression analysis to identify the risk factors for clinical LF. Of the 37 cases enrolled, 23 died (62% case fatality rate). Cases were younger than controls (19.5 years vs 28.9 years, *p* < 0.05) and more frequently female (64.8% vs 52.8%). Compared to the controls, clinical LF cases had higher contact with rodents (rats or mice) in their households in the preceding three weeks (83.8% vs 47.8%). Households with a cat reported a lower presence of rodents (73% vs 38%, *p* < 0.01) and contributed to a lower rate of clinical LF (48.6% vs 55.7%) although not statistically significant (*p* = 0.56). The presence of rodents in the households (matched adjusted odds ratio (mAOR): 11.1) and younger age (mAOR: 0.99) were independently associated with clinical LF.

Rodent access to households and younger age were independently associated with clinical LF. Rodent access to households is likely a key risk factor for clinical LF in rural SL and potentially in other countries within the West African region. Implementing measures to control rodents and their access to households could potentially decrease the number of clinical LF cases in rural SL and West Africa.

## Introduction

Lassa fever (LF) is a viral zoonotic illness caused by *Lassa mammarenavirus* (LASV), and is responsible for severe haemorrhagic fever characterized by muscle aches, vomiting, chest and abdominal pain, and bleeding from the mouth, nose, eyes and other mucous membranes with several complications including deafness [1]. The disease is endemic in West Africa including Sierra Leone (SL) [2–6]. In a 1980s estimate, LF was reported to infect approximately 200,000–300,000 people and cause 5,000–10,000 human deaths each year in West Africa [7]. However, in the last four decades, the population in sub-Saharan Africa (SSA) has doubled and crop production has intensified, resulting in losses of forest areas and destruction of ecosystems, which could have created conditions more favourable for LASV infection. A 2020 model estimated an annual incidence of more than 800,000 LF cases in West Africa [8].

*Mastomys natalensis* is the primary reservoir of LASV [9, 10]; however, two other species, *Mastomys erythroleucus* and *Hylomyscus pamfi*, were recently identified as reservoirs of LASV [11, 12]. Programmes on rodent control to fight against LASV conducted in West Africa listed several drawbacks in the successful elimination of rodents including the prolificacy of *M. natalensis* with a mean litter size of 9.2 (range: 3–14), the availability of alternate food that helps rodents to escape baited food, the porosity of the houses/rooms allowing the rodent to enter and live, and the low number of natural predators of rodents in the community [13].

In SL, most of the towns and villages are embedded in fragmenting forest or bush environments, creating opportunities for the invasion of species able to adapt to human conditions and housing. Most dwelling houses in SL store primary crops, and their residues from subsistence agriculture provide an easy food source to increase the likelihood of human contact with rodents and their faeces or urine.

Humans are believed to get infections by touching objects contaminated with rodent urine, breathing aerosolized particles, being bitten by rodents, or consuming rodents [14–16]. Human-to-human transmission can occur occasionally in hospital settings and the community [7, 17, 18]. Earlier studies identified several risk factors mostly associated with human-to-human transmission [19]. Kerneis et al. (2009) reported living with someone with haemorrhagic fever and receiving an injection in past years as a risk factor for LASV infection [19]. Another study from Nigeria reported that the LF cases had a history of consuming rodent-contaminated food (56%) or being exposed to LF-infected individuals (15.8%) [20].

Risk factors related to human-to-human infection further mean that the enrolled cases were not index cases. Furthermore, most of the risk factors identified were reported through a cross-sectional study, thus raising the ambiguity of the temporality of the instances and exposure. Nonetheless, no risk factors for clinical LF are reported in SL. Therefore, this study aimed to estimate the risk factors for clinical LF in an endemic district of SL to synthesize evidence to support policies and programmes to prevent household-level exposure to LASV in humans.

## Methods

We collected the list of LF cases identified between January 2019 and December 2021 from the national LF surveillance unit based in Kenema Government Hospital (KGH), SL. Our team consists of a research officer and a research assistant. Both received training on the administration of the questionnaire. The questionnaire was pre-tested in a similar village in the Kenema District and modified based on the field observation. We defined a case as a person who has been confirmed with presented positive results for LASV detection by either real-time reverse transcription–polymerase chain reaction (RT-PCR) or serology (IgM) with an illness consistent with a clinical description of known LF cases. Some cases were also recorded from Medecins Sans Frontieres (MSF), Hanga Town, Kenema District. Details of the laboratory testing of LASV are described earlier [3, 21, 22]. We defined a person as a control who lived within a one-kilometre radius of the case household and who had not shown any symptoms compatible with clinical LF in the past 3 weeks [23].

### Inclusion and exclusion criteria

***Cases:*** Inclusion criteria for the cases were as follows: (i) individuals with a confirmed positive test for LASV (RT-PCR or IgM), (ii) identified through KGH or MSF surveillance, and (iii) those who provided informed consent, or whose guardian/proxy provided consent for participation in the study. Patients with inconclusive lab results (e.g. only IgG positive) or those who did not provide consent were excluded from the study.

***Controls:*** Inclusion criteria for controls were as follows: (i) residing within a 1-km radius of the case household; (ii) no known clinical signs resembling LF, including fever, malaise, headache, sore throat, muscle pain, vomiting, nausea, diarrhoea, or haemorrhage, within 3 weeks before or after the identified LF case; and (iii) provided consent to participate in the study. Individuals with a history of clinical LF infection or those who tested positive for LF (IgM, IgG, or RT-PCR) at any point in their lifetime were excluded. Those who did not provide consent were also not included in the study.

***Sample size estimation:*** We estimated the sample size based on an expected odds ratio (OR) of 4.0, an assumed exposure rate of 18% in the control group [19, 20], a 95% confidence interval (CI), and 80% power. The calculated sample size was 40 cases. With a case-to-control ratio of 1:4, we anticipated enrolling a total of 160 controls.

**Figure 1.**
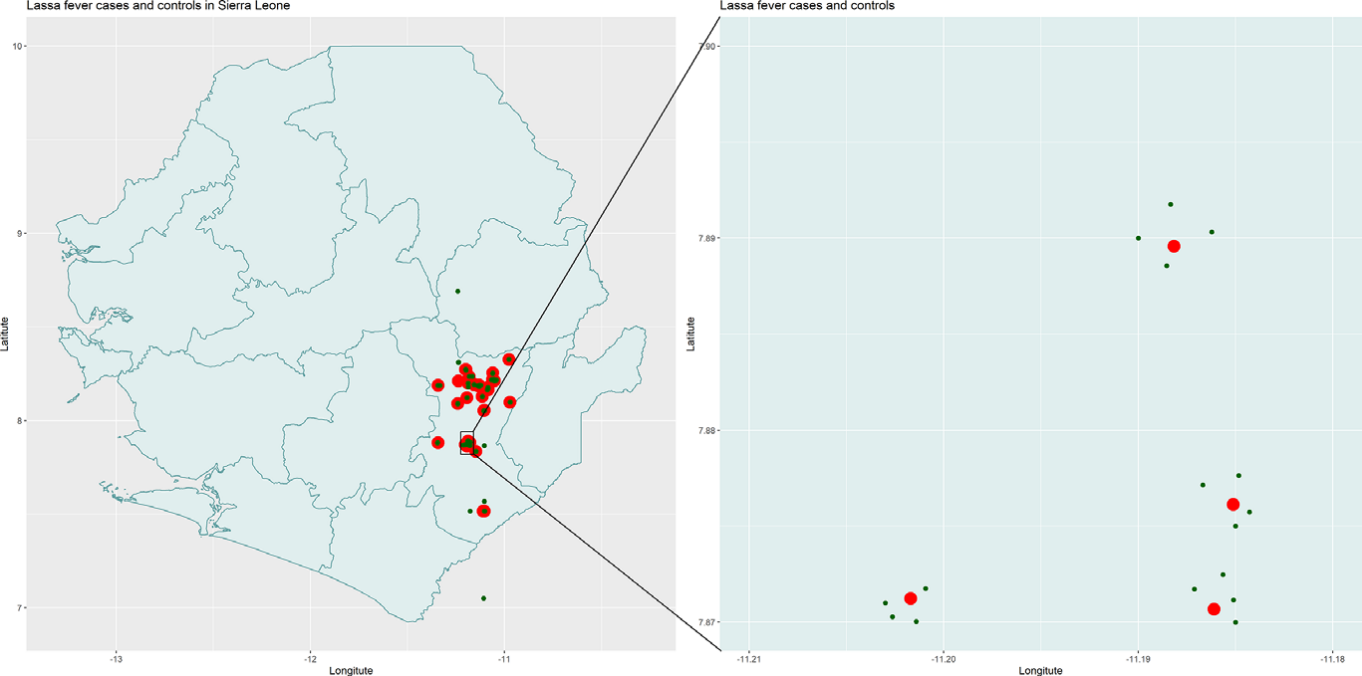
Map of Sierra Leone showing the location of clinical Lassa Fever cases and their healthy controls in Kenema District. For each case patient, four healthy controls were enrolled within one kilometre of the case household.

**Figure 2.**
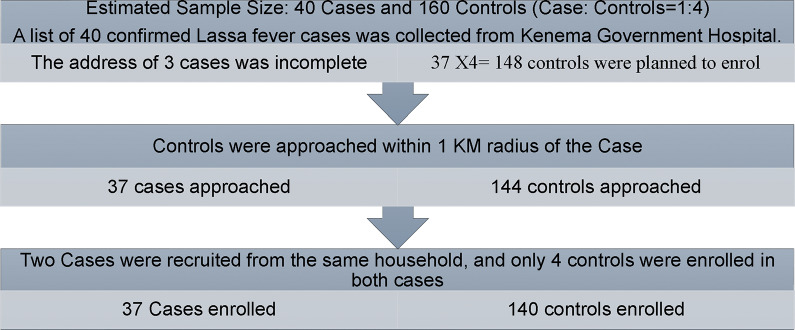
The flowchart of enrolment of clinical Lassa Fever cases and controls from Sierra Leone Clinical LF cases were tested positive between January 2019 and December 2021 in Sierra Leone. Data on cases and controls were collected between June 2021 and January 2022.

Between June 2021 and January 2022, we enrolled cases and controls from Kenema districts (Figure 1). We collected the lists of suspected LF patients for the period of January 2019 and December 2021. The list was provided by the head of the ‘Outreach Team lead of the LF unit’ of KGH to support the doctoral research of the first author (DJS). The database we reviewed contains 76 suspected LF cases, of which 40 were confirmed positive (RT-PCR and/or IgM enzyme-linked immunosorbent assay (ELISA)). We were able to enrol 37 cases, as the remaining individuals could not be located based on the addresses provided by KGH or MSF. After reaching the case’s house, we explained the objective of our study and requested a signed consent. If the case had died, we collected the data from the closest person related to the deceased person during their illness. In most cases, the closest person was one of the parents or siblings. The step-by-step method of enrolment of cases and controls is shown in the flowchart (Figure 2).

After obtaining written informed consent, we conducted interviews with the cases or the closest person of the case using a structured questionnaire with 51 questions, 11 of which included multiple sub-questions. The team inquired about the demographic information of the case (age and sex) and their exposure history in the 3-week before the onset of illness including the presence of rodents (rats or mice) in their households, rodents’ activities, having animal contact, presence of cats and dogs at households, involvement with bushmeat (hunting, processing, or eating), palm juice processing, and the physical location of the household including the estimated number of palm trees around 500-m radius of the case house. We recorded the location of the case house by obtaining their coordinates using handheld global positioning system devices.

For each case, the team enrolled four individuals as controls from a 1-km radius of the case’s location. We walk in each of the four directions from the case house (north, south, east, and west). From each direction, we enrolled one control randomly. After the agreement and signing of the written consent, we administered the same questionnaire used for the case. In one instance, two cases were enrolled from the same household, and we enrolled only four controls from them.

Individuals were excluded as controls if they had tested positive for LASV-specific antibodies (IgG or IgM) or PCR in their lifetime or had clinical signs/symptoms compatible with LF infection including fever, malaise, headache, sore throat and muscle pain, vomiting, nausea and diarrhoea, and haemorrhage in 3 weeks before and after the LF case was identified. In case the approached control was not enrolled, we walked in the same direction to identify another individual.

### Variables of interest

1. ***Exposure to*** ***rodents:** M. natalensis* mice are known reservoirs of LASV. We hypothesized that the presence of rodents and increased interaction with rodents will increase the risk of LASV infection. During our pre-testing of the questionnaire, we identified that people cannot differentiate between rats and mice, and for that reason, we used local language and description of each species to understand the exposure to mice and rats. We combined rats and/or mice into a single variable named ‘rodents’. Collectively, we had eight questions regarding exposure to rodents and rodents’ activity in their household including the presence of rodents (either rats or mice), frequency of rodents observed, and contact with rodents (touched, eaten, or processed).
2. ***Exposure to animals:*** We were interested in understanding whether contact with other animals might be associated with LASV infection and thus included questions on exposure to peri-domestic and domestic animals including monkeys, dogs, squirrels, bats, sheep, goats, cattle, and chicken.
3. ***Bushmeat:*** Bushmeat has been considered as a practice associated with the spillover of several zoonotic pathogens. We asked whether individuals were involved in hunting wild animals, processing wild animal meat, and the business of wild animals or meat.
4. ***Infected human:*** We hypothesized that contracting a LASV-infected individual would increase the risk of clinical LF and thus asked whether the subjects were exposed to LASV-confirmed cases 21 days before the onset of illness of the case individual.
5. ***Palm tree and palm juice:*** Palm tree or juice (*Poyo*) is not known to be associated with LASV infection. However, the presence of palm trees around the household may be linked to an increase in rodents in the area [24]. Also, rodents, especially squirrels or occasionally mice, can contaminate the palm juice collecting pot. Thus, we hypothesized that people involved with palm juice collection, processing, and business are at increased risk of clinical LF.
6. **Demography:** A large proportion (~80%) of LF cases are mild and asymptomatic [23], and lifetime cumulative exposure to LASV might act as a protective factor for the older population. We hypothesized that being younger in age and female increases the risk of clinical LF [23].
7. **Presence of cat(s) in the household:** Cats are reared to control rodents in households. We hypothesized that having a cat in the household would reduce the burden of rodents in the household and thus contribute to reducing the risk of clinical LF.
8. We have dropped a variable from the final multivariate logistic regression model if the variable (a) had less than 10% response, (b) had temporal ambiguity, and (c) was not biologically plausible.

## Data analysis

**Figure 3.**
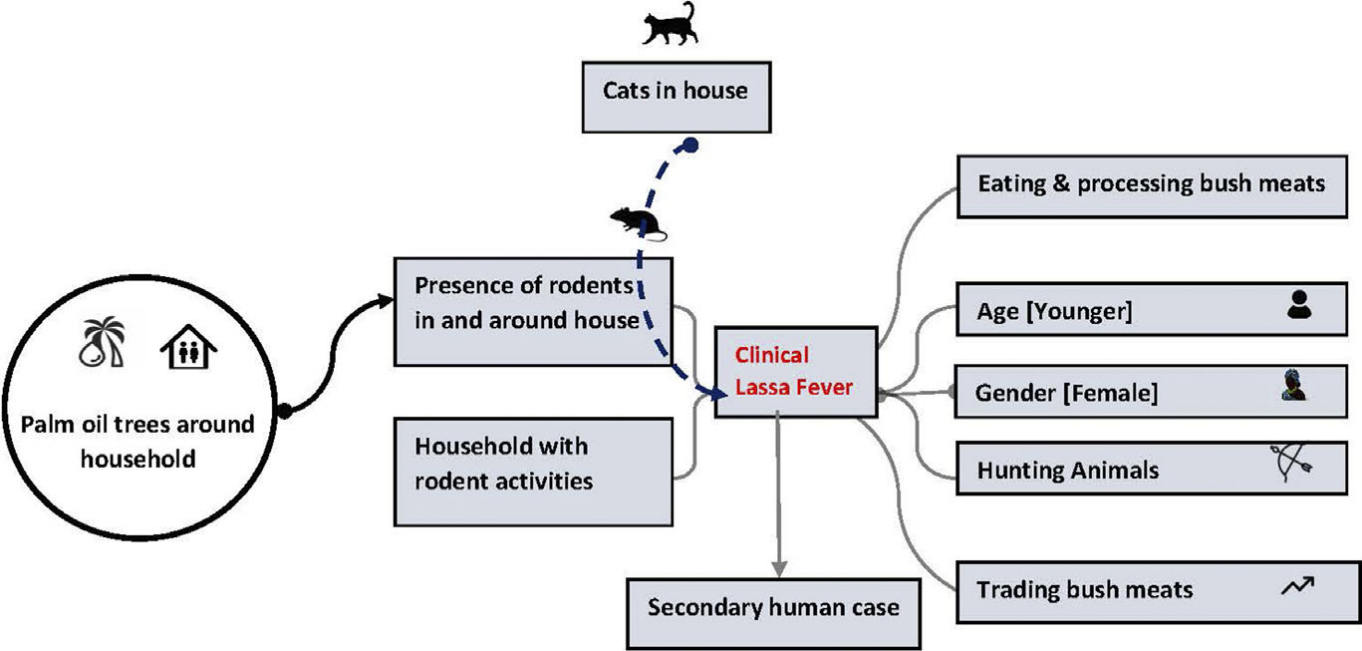
Hypothetical causal relationship between different biological and environmental factors (variables) and clinical Lassa Fever in Sierra Leone. A solid line indicates a direct relationship between variables. A higher number of palm oil trees are probably associated with the presence of a higher number of rodents in the neighbourhood, which ultimately results in the presence of rodents in households. The dotted line indicates interference with other variables. For example, the presence of cats in the house could control the number of rodents in the households and thus could reduce the risk of clinical Lassa Fever.

We reported numbers and percentages for categorical variables. For continuous variables, we used mean with interquartile range (IQR) or standard deviations. We performed a univariable analysis of variables for reporting the ORs and the 95% CI using logistic regression. To build the final regression model, we developed a hypothetical causal diagram by including the variables that are biologically plausible to cause clinical LF (Figure 3). We included eight variables that were biologically plausible in the conditional multiple logistic regression model irrespective of their significance in univariate analysis to estimate adjusted matched ORs and 95% CI. We included only one rodent exposure-related variable (presence of rodent-related exposure in the household) in the final model as other variables indicating the degree of exposure to the households (e.g. frequency of observing rats and mice (1–2 times vs more than) or rodent activity at the house (observed rat holes, nest, droppings, pups, and food damage by rodents). None of the comorbidities (diabetes, hypertension, and arthritis) was eligible for inclusion in the model (with more than 50% missing responses). The data analysis was performed in the statistical software STATA version 17. Conditional logistic regression analysis was conducted using the ‘clogit’ function by including all controls of each case as group variables.

**Ethical approval:** This study was approved by Sierra Leone Ethics and Scientific Review Committee on 31 October 2019 and the Clinical Research and Ethical Review Board of the Royal Veterinary College, University of London, United Kingdom, on 27 March 2022 (URN 2019 1949-3).

## Results

We enrolled 37 clinical LF cases and 140 eligible controls. Of the 37 cases, 23 died of the infection, indicating a case–fatality ratio of 62%. The mean age of the deceased cases was 17.0 (IQR: 3.3–24.0) years, while the mean age of the survivors was 21.1 (IQR: 11.5–28.0) years. Of the 37 cases, 36 were hospitalized, 33 had fever, 28 had body aches, 21 had joint pain, 11 had vomiting, 10 had coughing, and 4 had bleeding from natural orifice. On average, clinical LF patients stayed 11.6 days (IQR: 7–14) in the hospital before discharge or death, with survivors staying an average of 12 days (IQR: 7.0–13.5) and those who died staying 8.7 days (IQR: 5.5–9.2). None of the cases or controls had visited another confirmed clinical LF or visited any hospital 21 days before the onset of illness of the case patient. Except for one control respondent, all participants have heard of the term ‘Lassa Fever’.

More than 64% (*n* = 24) of the cases and 52% (*n* = 74) of the controls were female. Compared to the controls, the cases were younger (19.4 vs 28.8 years, *p* = 0.01). Cases reported the presence of rodents (rats or mice) more frequently than the control in the household in the past 3 weeks (83.8% vs 47.8%, *p* < 0.01). Case also observed a higher frequency of daily observation of rodents in the household (72.9% vs 40.7%, *p* < 0.01) (Table 1). Cases and controls did not differ in terms of exposure to wild meats including hunting, processing, eating, and/or trading (18.9% vs 24.2%, *p* = 0.63) or having a cat in the household (48.6 % vs 55.7%) (Table 1). We also explored the relationship between several exposure variables including households with cats and reporting rodent activities. Of the 96 households that reported having a cat, only 38% (*n* = 38) observed rodent activities in their household compared to 73% (*n* = 58) without any cat in the household (*p* < 0.001).

**Table 1.** Demographics and other important variables of clinical Lassa Fever cases vs control individuals in the Kenema District of Sierra Leone identified from January 2019 to December 2021

	Variables	Cases (%) (*N* = 37)	Controls (%) (*N* = 140)	*P*-value	Matched OR (95% CI)
1	Age of subject in years and mean (standard deviation)	19.5 (±18.8)	28.9 (±20.8)	<0.01	0.993 (0.989–0.996)
2	Female gender (%)	24 (64.8%)	51 (52.8%)	0.29	1.5 (0.71–3.2)
3	Presence of rodents (rats or mice) in the household in the past 3 weeks	31 (83.8%)	67 (47.8%)	<0.001	6.8 (2.5–18.6)
4	Frequency of observing rats and mice (1–2 times vs more than twice daily)	27 (72.9%)	57 (40.7%)	<0.001	3.9 (1.81–9.12)
5	Rodent activity at the house (observed rat holes, nest, droppings, pups, and food damage by rodents)	23 (57.5%)	57 (40.7%)	0.01	2.6 (1.2–5.9)
6	Having a domestic animal contact (processing, killing, or cooking animals) in the past 3 weeks	25 (67.6%)	86 (61.4%)	0.60	1.60 (0.61–4.20)
7	Touching of wild or peri-domestic animals’ animals (mice, rats, monkeys, squirrels, or other wild animals) in the past 3 weeks	7 (18.9%)	11 (7.8%)	0.09	2.7 (0.84–9.9)
8	Presence of a cat in the household	18 (48.6%)	78 (55.7%)	0.56	0.75 (0.36–1.55)
9	The mean number of palm oil trees around 100-m radius of the household	9.75	4.10	0.36	1.03 (0.98–1.13)
10	Exposure to bushmeats (hunting, eating, processing, and trading bushmeats)	7 (18.9%)	34 (24.2%)	0.63	0.92 (0.29–2.91)
11	Any member of your family collected palm oil juice	15 (37%)	38 (27.1%)	0.16	1.83 (0.84–3.87)
12	Presence of the dog in the household	5 (13.5%)	21 (15.0)		1.06 (0.36–3.12)

The multivariable analyses provided evidence of an association between the odds of LASV infection and the presence of rodents in the household (matched adjusted OR (mAOR): 11.1 (95% CI: 2.8–42.4)) and age in years (mAOR: 0.99 (95%: 0.98–0.99)) (Table 2). Other variables, including gender, showed no evidence of association with the odds of infection following adjustment for other variables (Table 2).

**Table 2.** The factors associated with clinical Lassa Fever in humans in a multiple logistic regression analysis in Sierra Leone, 2019–2021.

Risk factors	Matched Adjusted Odds Ratio (mAOR)
Female gender	1.15 (0.45–2.98)
Age of the subject	0.99 (0.98–0.99)
Presence of rodents (rats and mice) in the household in the past 3 weeks	11.1 (2.8–42.4)
Exposure to wild animals or bushmeat	2.87 (0.56–14.6)
Touching wild animals (mice, rats, monkeys, squirrels, or other wild animals) in the past 3 weeks	4.18 (0.66–26.1)
Having a domestic animal contact (touching, processing, killing, or cooking animals) in the past 3 weeks	0.86 (0.20–2.60)
Having cats on the housing premises	0.50 (0.17–1.39)
Dogs at household	1.84 (0.41–8.26)

## Discussion

We identified rodent access in the household markedly increased (e.g. by 11 times) the risk of clinical LF in humans in rural SL. We further found that the younger the individual the higher the risk of developing fatal LASV infection. In the univariable analysis, we observed a dose–response relationship with rodent activity: seeing rodents more than twice, compared to 1–2 times, was associated with an increased risk of clinical LF (AOR: 3.9). Furthermore, the daily observation of rodent activity at a higher frequency was associated with an increased risk of clinical LF (AOR: 2.6). This is highly plausible and supports our current understanding of LASV transmission in rural West African settings.

The multimammate mouse, *M. natalensis*, has been considered the key reservoir of LASV, with humans being infected directly or indirectly through fluids of the mice such as urine, saliva, and blood [25]. A previous study conducted at our field sites in SL found that 92% of residents reported the presence of rodents inside their households, and 57% of the trapped rodent species were identified as *M. natalensis* [25]. A recent rodent trapping study in the same areas identified 2.8% of trapped *M. natalensis* tested positive for LASV [26], highlighting a significant risk of rodent–human transmission.

LASV has been circulating in West Africa for the past six decades, or possibly even hundreds of years, posing a continuous public health threat to the region. However, the identification of risk factors for LASV infection or clinical LF through case–control studies is extremely rare. One possible obstacle to such a study is that LASV infection, when clinically manifested, is very severe and often fatal [23], and collecting data from the cases is challenging. Another potential barrier is that a vast majority of the cases are asymptomatic [23], making case enrolment difficult and increasing the risk of misclassification without laboratory confirmation. Nevertheless, a case–control approach has proven to be ideal when knowledge of potential risk factors is limited, allowing for the investigation of a wide range of risk factors associated with different causal pathways. Our study, despite some of these existing limitations, attempted to identify risk factors for clinical LF and helped generate several hypotheses that need further systematic research.

Several cross-sectional studies established the link between exposure to rodents and LASV infection. A study conducted in rural Guinea in the 1990s identified hunting peri-domestic rodents and consumption of rodents as potential risk factors [16]. Another study further identified household-level risk factors for increased abundance of rodents, including households having more than 8 holes and the presence of rodent burrows [27]. Thus, our findings support the current understanding of household-level rodent–human LASV transmission. In our enrolled study population, none of the cases reported visiting a hospital or sick people 21 days before the onset of illness indicating a primary spillover of the LASV infection.

We found that younger subjects are more exposed to LASV and develop clinical LF. Furthermore, the deceased cases were younger than the survivors (17.0 years vs 21.1 years). A large proportion of LASV infections are asymptomatic [23], and thus, older people possibly acquire immunity against clinical LF through lifetime cumulative exposure to the virus.

Although the final multivariable analysis did not provide evidence of other variables being associated, our study raised several potential hypotheses. For example, cats have been promoted in rodent-killing programmes in West Africa but whether the cats can reduce the burden of rodents or become infected themselves and be a source of transmission has not been studied. In our univariate analysis, we found that households with a cat reported lower rodent activity on the premises (73% vs 38%, *p* < 0.05) and had a reduced proportion of clinical LF (48.6% vs 55.7%), although this difference was not statistically significant (*p* = 0.56). However, this could be an economic artefact, as the presence of a cat in the household may reflect greater economic stability, which could lead to better housing conditions that limit rodent access. Ideally, the association between two exposure variables is viewed as a confounder. However, we included both variables (rodents and cats) in the final regression model, as each could influence LASV exposure. It would be valuable to explore further how the presence of cats (or the number of cats) in households may reduce rodent infestations to a level sufficient to control clinical LF. Our study also indicated that households with clinical LF cases had a higher number of palm oil trees within a 500-metre radius. While the palm tree itself is not a direct risk factor, the increased presence of palm trees may create a more conducive environment for rodents nesting in the surrounding bushes. Future research should investigate the potential contamination of palm tree juice (locally known as ‘*Poyo*’) collected from oil palm trees for evidence of LASV.

Our study found no increased risk of clinical LF associated with exposure to bushmeat, the presence of dogs in households, or family members’ involvement in palm tree juice (*Poyo*) preparation or related businesses. However, the lack of evidence in our study does not necessarily exclude these variables as potential risk factors for clinical LF in other settings or a well-designed study conducted in the same context. Some of these variables have been identified as risk factors in other countries, and the statistical power of our study was limited due to the small sample size [16].

This study has several limitations. First, we did not confirm the controls as test negative. This is critical when we know that a large proportion of LASV infections are asymptomatic and people living in endemic areas like Kenema District might have a high prevalence of LASV exposure (e.g. 20.1%) [22]. We tried to minimize potential classification bias by asking for all the clinical signs compatible with clinical LF. As LASV is a serious concern in the community, we believe people pay attention to their illness when a case of LASV is identified in the community. All our controls were enrolled from the same community, within a 1-km radius of the case individual. However, our study could not adjust for possible misclassification due to asymptomatic infection among controls. Therefore, the risk factors we report should be interpreted as specific to clinical LF, not to LF infection in general. Second, like all other case–control studies, our study might have included recall bias. To avoid recall bias, we physically verified some of the questions. For example, access to rodents in the households was observed and questions were placed in a way that the respondent could self-verify his response. Thus, we believe recall bias was minimal in our study. Finally, we took verbal autopsies of the cases who died of LASV infection, which might lead to some information bias. However, most questions we included were answerable by any nearest individuals as most LASV exposure is household level (e.g. rodents’ access to household) or through group exposure (e.g. bushmeat).

## Conclusion

The presence of rodents in the households (mAOR: 11.1) and younger age (mAOR: 0.99) were independently associated with clinical LF. Rodent access to households is likely a key risk factor for clinical LF in rural SL and potentially in other countries within the West African region. Implementing measures to control rodents and their access to households could potentially decrease the number of clinical LF cases in rural SL. Vaccines when available should target the younger-aged population as a priority. We recommend studying the role of cats in the prevention of rodents, thereby reducing the overall risk of clinical LF in endemic countries.

## Data Availability

All data used in this study were collected through face-to-face interviews conducted by the field research team with the participants. Anonymous data can be made available upon request from the corresponding author.
